# Estimating the Depths of Normal Surface Notches Using Mode-Conversion Waves at the Bottom Tip

**DOI:** 10.3390/s24154849

**Published:** 2024-07-25

**Authors:** Qianghua Pan, Jiawei Xu, Wenbo Li, Haiyang Li, Zehui Li, Pengfei Wang

**Affiliations:** 1 China Special Equipment Inspection and Research Institute, Beijing 100029, China; pqh23@163.com (Q.P.); lwb123@163.com (W.L.); 2 Key Laboratory of Advanced Manufacturing Technology, North University of China, Taiyuan 030051, China; xurefl@163.com (J.X.); lzh_2000@foxmail.com (Z.L.); pengfeiwangwork@foxmail.com (P.W.); 3 Shanghai Acoustic Laboratory, Chinese Academy of Sciences, Shanghai 201815, China

**Keywords:** Rayleigh waves, surface crack, crack depth measurement, ultrasonic testing

## Abstract

In this work, a two-parameter inversion problem is analyzed, related to surface crack widths for measuring depths of normal surface notches, based on a laser-based ultrasonic measurement method in the time domain. In determining the depth measurement formulas, the main technique is the time delay between reflected and scattered waves. Scattered waves are generated by two reflections along the bottom and three mode transformations at the surface of the crack tips. Moreover, the scattering angle of the mode-conversion waves is 30°. These two key factors lead to corrected item “2wβ” in the depth measurement formula. A laser-based ultrasonic experimental platform is built to generate and receive surface waves in a non-contact manner on aluminum and steel specimens with surface cracks. The depth measurement method proposed in this paper has been validated through theoretical, simulation, and experimental methods. Finally, in this paper, an effective approach for quantitatively measuring crack depths, based on laser ultrasound, using the time-domain properties of surface wave propagation is provided.

## 1. Introduction

Surface cracks, which are usually the start of deeper defects in materials after expansion under external loads, are not promptly detected, leading to considerable safety hazards of materials. Surface cracks usually have two dimensional parameters, such as depth and width, which are key parameters that directly affect the propagation speed of micro-defects and macro-defects. For quantitatively determining crack depths, it is crucial to accurately evaluate the material properties [1,2]. Rayleigh waves, used for measuring surface crack depths, have been widely researched for several decades [3,4,5]. Traditional non-destructive techniques, relying on transducer contact coupling on the specimen surface to generate and receive Rayleigh waves to detect surface cracks, are limited by the condition of the severe environment of detected specimens [6]. To overcome this difficulty, increasing attention is being paid to non-contact non-destructive detection techniques, such as electromagnetic ultrasonics [7], air-coupled ultrasonics [8], and laser-based ultrasonics [9]. However, both electromagnetic and air-coupled ultrasonics are still restricted by the performance of transducers; in particular, electromagnetic ultrasonics are only suitable for metallic materials. According to the reasons above, the laser-based ultrasonic technique is chosen as detection tool in this work because of its advantages, such as high sensitivity and contactless detection.

Laser-based ultrasonic methods can be divided into different means of measurement based on reflection [10], transmission [11], and mode-conversion phenomena [12], which happen at the front and back faces and tips at the bottom of surface cracks. In addition, from the perspective of acoustic signal processing, laser-based ultrasonic methods are also divided into time [13], frequency [14], and time–frequency measurement methodologies [15]. And according to the ratio of crack depth to incident waves’ wavelength, for surface cracks with depths shorter than the wavelength of the incident surface waves, sub-wavelength depth measurement methods based on laser ultrasound are isolated among other methods [16], because the whole range of the crack depth is covered with the Rayleigh surface wave measurement method. Bernard [17] pointed out that the time method is suitable for cracks for which the ratio of crack depth to incident waves’ wavelength is higher than 0.8; in this case, the crack depth is deep enough to distinguish the arrival times of reflecting, transmitting, and scattering waves at cracks. However, for ratios lower than 0.8, the frequency technique, which makes use of the different reflection and transmission abilities of waves having different wavelengths, is more sensitive. That is to say, measurement limitations exist for measuring crack depths both by the time method and the frequency method. Distorted behaviors in the frequency domain are observed as central frequency shifting [18] and the symmetric spectral loss caused by the fast reduction in the low-frequency or high-frequency components’ amplitude [17]. This is a common way that the curves of reflection and transmission coefficients related to crack depths are built to quantitatively measure cracks [19]. The parameter variation in the frequency domain is so sensitive to sub-wavelength cracks that micro-cracks with depths comparable to the magnitude of material grains can also be quantitatively measured. The influences of cracks on wave propagation and the indirect relationship between surface waves and frequency were discussed in [20].

Li [16] observed a threshold called the critical frequency, and he found that reflection and transmission wave energy conversion exists in the frequency domain and that the critical wavelength corresponding to the critical frequency is approximately equal to four times of crack depth. Finally, this method successfully estimate the crack depth, even though its detection ability is limited by the frequency bandwidth of laser ultrasonics, just like other measurement methods in the frequency domain. Reda [21] discusses the propagation characteristics of waves in periodic structures and points out that changes in cracks may affect the relationship between the surface waves and frequency.

Utilizing the time characteristics of surface wave propagation to detect the depth of surface defects is another reliable method. In the ultrasonic time method, the time delays of different waves propagating along the crack surface are a key parameter for determining crack size. Therefore, provision of the wave paths of different waves at cracks is the first procedure for measuring crack size. Shan [22] successfully investigated a laser TOFD technique to determine the cracks’ depths in a through-thickness direction by calculating the arrival time of a mode-converted wave at the tip of the fatigue crack. Jian [23] proposed a gauging method based on an analytical calculation formula of the arrival times of reflection, transmission, and mode-conversion waves using the finite difference method. Cooper [18] proposed an analytical formula based on the time difference between reflected and scattered Rayleigh waves to measure crack depths ranging from 0.1 to 0.5 mm. Even though the experimental data verified the validity of this quantitative temporal methods, the two problems mentioned in this work remain unsolved. One of the issues pertains to the influence of the crack width on the measurement of the rectangular notch depth. In order to obtain more accurate depth values, Cooper adjusted the analytical formula by incorporating twice the crack width. However, the authors of this study did not provide a comprehensive rationale for this adjustment, and this aspect has been overlooked in other publications in the field. For instance, Jeong [24] conducted depth measurements for defects ranging in depth from 0.3 mm to 5 mm in an experiment, but did not consider the width influence of rectangular notches. The results indicated that the measurement errors for cracks with depths of 0.3 mm and 1 mm were larger than those for other cracks. Jeong failed to account for the variations in errors across all crack measurements, as the first-order fitting curve was utilized to assess crack depth, thereby concealing this phenomenon. Li [25] attempted to provide a theoretical explanation for the empirical formula in set out in [18]. However, Li’s work in [25] did not focus on the wave propagation path on the surface notch interfaces, but rather relied on the formula in [18]. Consequently, the theoretical work in [25] requires reconstruction and validation using more precise experimental data. Another issue with the evaluation formula proposed in [18] is the lack of evidence for why the propagation angle of the scatter mode acoustic wave at the tip of the rectangular notch is assumed to be 30°. The scattering angle at the tip of rectangular notch holds significant importance in predicting crack depth, similar to the influence of crack width. As mentioned earlier, the detection capability of the frequency spectrum method is suitable for short cracks and is associated with the ratio of crack depth to the wavelength of the incident wave. In terms of time detection, the impact of crack width on crack depth measurement cannot be disregarded, and shorter cracks lead to greater measurement errors. Therefore, further research is directed towards investigating the interaction between Rayleigh waves and surface cracks, taking into account the influence of crack width on wave propagation.

In this presentation, the depth of a normal surface notch is estimated by considering crack width and utilizing mode conversion wave at the bottom tip. The rectangular notch, created by wire-electrode cutting, is studied as a surface crack due to its ideal tips for detection. The depth/width ratio of the notch is used to categorize it into three types: extremely narrow, narrow, and wide defects. To gauge the depth of these rectangular notches, the arrival time and propagation path of the reflected wave and mode-converted wave at the tip of the rectangular notch are utilized to develop an analytical formula corrected by the width of the notch. The simulation results obtained through COMSOL Multiphysics 5.5 software and experimental data effectively validate the proposed theoretical analysis. The main content contains four parts: (1) the propagation paths of the reflected and transmitted and mode-converted waves at rectangular notch are presented, and the analytical formula for the extremely narrow, narrow, and wide rectangular notches are built in Section 2.1; (2) the scattering angle at the tip of the rectangular notch obtained by the acoustic field simulated by the COMSOL Multiphysics software and the proposed measurement formula for three types of rectangular notch are verified in Section 2.2, Section 2.3, Section 2.4 and Section 2.5; (3) the optical experimental platform based on the line focus method and thermal elastic effect is built, the acoustic wave signals are acquired by the auto scanning platform to size the rectangular notch depth using the proposed prediction formula in Section 3; (4) finally, the discussion and conclusion of the simulation and experimental results are presented in Section 4.

## 2. Theoretical Analysis and FEM Simulation

### 2.1. Theoretical Analysis

**Figure 1:**
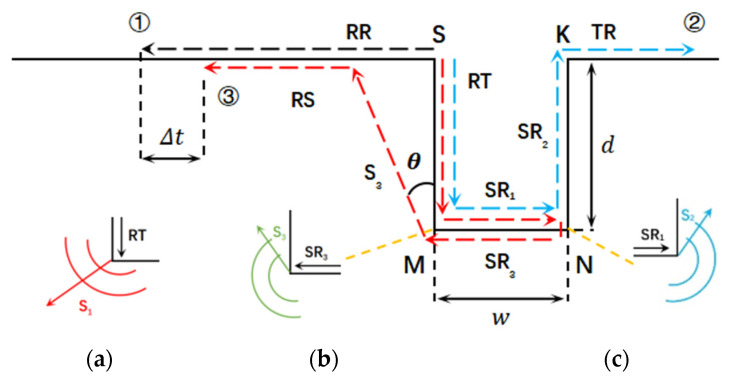
Tip waveform conversion phenomenon at surface defects. (**a**) The first mode conversion; (**b**) The third mode conversion; (**c**) The second mode conversion.

In the two-dimensional plane, the propagation pattern of Rayleigh surface waves manifests as a diminishing ellipse in the direction normal to the surface. The amplitude of these waves diminishes rapidly with distance from the surface, following an exponential decay model, leading to concentrated energy within the material to a depth nearly twice the wavelength. This characteristic enables the Rayleigh surface wave technology to assess the surface integrity of specimens over long distances. When the Rayleigh surface wave meets discontinuous interfaces, reflection, transmission, and mode conversion waves are generated, which can be used to determine the crack size by the change in the time and frequency domains. The rectangular surface notch is a structure made of two interfaces, one finite length bottom, and two tips. Figure 1 depicts the acoustic file generated at a rectangular notch with a depth of d and width of w, including the incident, reflected, transmitted, and tip-scattering waves, where RR is the reflected sound wave, TR is the transmitted sound wave, and RS is the surface wave formed by the shear wave signal S_3_ on the surface of the material.

In Figure 1, the rectangular notch is delineated by the lines SM and KN representing the two interfaces, with points M and N marking the tips, and line MN defining the bottom of the notch. To better elucidate the phenomenon of waveform conversion at the notch tip, the Rayleigh surface wave propagation process is categorized into three paths. The path ① is a reflection path along the surface of the specimen labeled with a black line. At point S, one portion of the incident wave continues as a transmitted wave along the crack surface, labeled as TR. Another portion reflects at the left interface of the crack and returns along the surface, denoted as RR. The remaining part consists of scattering waves at tip S, which are ineffective for crack sizing as they penetrate into the material and cannot be detected. The S-M-N-K path ② is a transmission path labeled with a blue line, where after the scattering at tip M and tip N, a direct Raleigh surface wave along the path SM-MN-NK arrives at another side of the notch resulting in the transmitted waves RT. In this procedure, the transverse wave S1 is scattering waves generated at point N. It is worth noting that the propagation direction of S_1_ and S_2_ are different but with the same scatter angle 30°and that on account of that, the propagation directions of RT and SR_1_ relative to the bottom MN are different. The zoomed pictures at the tip M and N are shown in (a) and (c), where the mode conversion phenomenon at two tips are presented. Path ③ is a mixed path of reflection and scattering waves along the bottom MN labeled with a red line. It can be seen that path ③ starts at point S and ends at point M, including three scatterings and one reflection. The scattering wave S_3_ is generated at the third mode conversion at point M in zoomed picture shown in (b). The wave S_3_ arrives at the specimen surface and is detected as Rayleigh wave RS. Transverse waves S_1_ are scattering waves generated by the Rayleigh wave RT and encounter the tip M when the Rayleigh wave RT propagates along the interface SM. This is the first mode conversion at the tip of notch. After that, the Rayleigh wave SR1, as part of the Rayleigh wave TR, continues to propagate along the bottom MN and meets the tip N resulting in the second scattering. The transverse wave S_2_ is generated at the tip M as the second mode conversion. Meanwhile, the reflected Rayleigh wave SR_3_ propagates in the opposite direction of the Rayleigh wave SR_1_ propagation. And then, the reflected Rayleigh wave SR_3_ encounters the tip M again resulting in the scattering wave S_3_ being generated due to the third mode conversion. Transverse wave S_3_ propagates at a directional angle of 30° and reaches the specimen’s surface, where it is detected as the surface wave RS. Using the arrival time delay between the reflected waves RR and the scattered wave RS at path ① and path ③, depth of defect d is evaluated by the time delay Δ*t* using the trigonometric function, as:

(1) $$d=\frac{(\Delta t-\frac{2w}{v_{r}})\cos\theta v_{r}v_{s}}{\left(\cos\theta -\sin\theta \right)v_{s}+v_{r}}=\frac{\Delta t\cos\theta v_{r}v_{s}}{\left(\cos\theta -\sin\theta \right)v_{s}+v_{r}}-\frac{2w\cos\theta v_{s}}{\left(\cos\theta -\sin\theta \right)v_{s}+v_{r}}$$

where ν_r_ and ν_s_ are the wave velocity of the surface wave and the shear wave, respectively; θ is the scattering angle of the shear wave S_3_ at the point M generated by the third modal transition; and w is the width of the notch. Assuming $\beta =\frac{\cos\theta v_{s}}{\left(\cos\theta -\sin\theta \right)v_{s}+v_{r}}$, β is a constant, which is related to ν_r_, ν_s_, and θ.

The estimation formula of defect depth can be further written as follows:

(2) $$d=\frac{\Delta t\cos\theta v_{r}v_{s}}{\left(\cos\theta -\sin\theta \right)v_{s}+v_{r}}-2w\beta$$

As can be seen in Formula (2), the proposed detection technique is based on the arrival time difference in the reflected wave at the left interface of surface notch and the scattering wave at the left tip of the surface notch. More important is that the detection precision of the notch depth is related to the width of the surface notch. In order to analyze the effect more clearly, three types of surface notches are discussed, namely the narrow defects, extremely narrow defects, and wide defects according to the defect size ratio w/d, which is the ratio of surface notch width to depth. Three different types of notches are presented as follows:

When the defect size ratio satisfies 0.05 < w/d ≤ 1, the surface notch is defined as the narrow defect, the effect of the surface notch width cannot be ignored. In other words, the term “2wβ” in Formula (2) cannot be ignored. Another form of defect size ratio in this case is 0.1 < 2 w/d ≤ 2 corresponding to the item “2wβ” in Formula (2). The defect size ratio is actually the ratio of the wave propagation distance at the bottom to the notch depth. If this ratio is a comparable value for the arrival time discrepancy of path ① and path ③ during the measurement procedure of sizing defect, this size notch is narrow defect and the item “2wβ” cannot be ignored. The upper and lower limits of the narrow defect size ratio are presented in Section 2.4, which were measured using the finite element method with COMSOL Multiphysics software.

(1) When the defect size ratio is w/d ≤ 0.05, it exceeds the lower limit of the narrow defect range, and the surface notch is defined as extremely narrow. In this case, the minimum width of the defect is less than one twentieth of its depth, and the bottom of the notch is so narrow that the defect appears as a partial crack, with the distance between its faces being negligible. The term “2wβ” denoting the effect of the surface notch width is ignored in Formula (2).
(2) When the defect size ratio is w/d > 1, it exceeds the upper limit of the narrow defect range, and the surface notch is defined as wide. In this situation for sizing defect, Formula (2) does not work for sizing the crack depth any more. The width of the defect is greater than the depth, which makes the impact of defect width on the defect depth measurement very large. This is also expounded in [18]. The surface defect depth cannot be quantitatively detected using the arrival time discrepancy of the reflected wave and scattered wave accurately, and the proposed detection method based on Formula (2) is no longer applicable. The detailed explanation for this is shown in Section 2.4.
(3) Formula (2) is also proposed by Cooper for sizing the surface notch in [18], where the detected defect is attributed to a narrow defect of case (1). Cooper identified that the last term relative to the width of the notch is a corrected term using experimental data and the scattering angle θ is 30°. The reason for is that at this angle, the measurement error is minimum after frequent attempts. Formula (2) is not reliable for application in the actual detection if there is insufficient explanation for these two problems. As mentioned in Section 1, the reasons for these two problems are given here. The reflected Rayleigh wave RR at path ① is directly reflected from the left surface of the defect, while the scattering Rayleigh surface wave RS at path ③ is produced by the tip of the notch after two scatterings at point M and N along the bottom of the notch. Then, a certain time difference is identified between the reflected surface wave RR in the receiving path ① and the tip scattering surface wave RS in the receiving path ③, and the time difference is related to the depth and width of the surface notch, which provides an idea for the quantitative detection of the geometric parameters of the surface defect. The reason for item “2wβ” in the formula is twice propagation at the bottom of the surface notch. The first propagation on the bottom is wave SR_1_ and the second propagation on the bottom is wave SR_3_. After that, the transverse wave S_2_ is generated at the tip M of the notch and is detected as wave RS. So, the time delay between path ① and path ③ is relative to two times of notch width. That two reflections happen at the bottom is the first reason for Formula (1) using the corrected term. Cooper [18] considers the wave S_1_ is generated by the scattering wave at the tip M of the notch due to wave RT propagation. So, Cooper’s developed analytical measurement formula did not have the corrected item of “2wβ”. The measurement errors are demonstrated by the experimental data.

Another reason for the corrected item “2wβ” is the scattering angle of the Rayleigh wave at the tip of notch. The first mode conversion at path ③ is the process in which the surface wave RT propagates along the defect surface and first encounters scattering at the M point. The scattered angle of the transverse waves generated at this tip is 30° in clockwise rotation along the direction of wave propagation. The tip M of the notch is seen as a new source in the process of scattering wave S1 generated at the tip. The propagation directivity of the transverse wave scattering at the tip is similar to that of the transverse waves based on laser. There is a popular belief [25] that the propagation angle of the transverse wave generated by laser is 30°. So, the scattering angle of the transverse wave at the tip is also 30°. The scattering wave S_1_ generated by the first mode conversion is not detected because its scattering angle leads to inner propagation in the material. The scattering wave S_3_ generated by the third mode conversion is detected as the wave RS due to the scatter angle shown in Figure 1b. Thus, the wave RS as received by Cooper is generated by the scattering wave S_2_ from the surface wave SR3, not by the scattering wave S_1_ from the wave RT. This is the cause why the corrected item “2wβ”, that is twice the notch width, cannot be omitted in Formula (2). Without considering the wave propagation attenuation, many scattering and reflections between the notched surfaces can occur. However, in practice, only the acoustic signal generated by the third mode conversion is detected.

### 2.2. Finite Element Simulation and Results

Due to the symmetry in space, where a surface rectangular notch runs through the specimen along one side and is parallel to the laser line source, the three-dimensional problem of the interaction of Rayleigh waves generated by laser and the surface notch is simplified into a two-dimensional problem. The software COMSOL Multiphysics is used to establish a two-dimensional model to simulate reflected, transmitted, and scattered acoustic fields at the surface notch. 

Since finite element simulation represents an idealized state, in order to compute the propagation of surface acoustic waves in the material, the damping effect during the propagation process is neglected. The finite element form of the transient heat conduction equation is expressed as follows:

(3) $$KT+CT^{\prime }={R_{Q}+R}_{q}$$

In the formula, K: thermal conductivity matrix of the test material;

C: heat capacity matrix of the test material;

T: temperature matrix of the test material;

T′: time-dependent temperature change rate;

R_q_: heat flux vector of the test material;

R_Q_: heat source vector of the test material.

Due to the acceleration theorem, the linear governing equation for the thermoelastic effect can be expressed as follows:

(4) $$MU^{\prime \prime }+KU=R_{ext}$$

In the formula, M: mass matrix of the test material;

U: vector displacement of the test material;

U″: vector acceleration of the test material;

R_ext_: thermal stress vector caused by transient temperature strain.

For a thermoelastic body, the thermal stress vector R_ext_ can be expressed as follows:

(5) $$\int_{Ω}B^{T}D\varepsilon_{0}dΩ$$

In the formula, ε_0_: initial strain of the object;

B: strain matrix;

D: material parameter matrix.

In the finite element calculation process, the laser heat source can be expressed as a function of laser irradiation in space and time, as well as the material reflectivity to laser irradiation. The expression for the pulse energy of a laser line source can be expressed as follows:

(6) $$q=E\left(1-R_{i}\right)f\left(x_{1}\right)g\left(t\right)$$

In the formula, $f\left(x_{1}\right)=\frac{1}{\sqrt{2\pi }}\frac{2}{R_{G}}e^{\frac{-2x_{1}^{2}}{R_{G}^{2}}}$,$g\left(t\right)=\frac{8t^{3}}{v^{4}}e^{\frac{-2t^{2}}{v^{2}}}$

E—the energy per unit length of laser pulse;

R_i_—surface reflectance;

R_G_—Gaussian beam radius;

υ—rise time of laser pulse.

**Figure 2:**
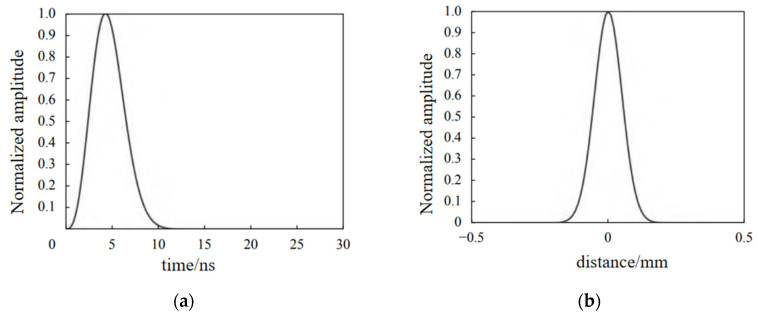
Time and space functions of laser source: (**a**) time function; (**b**) spatial function.

According to Table 1, in relation to the time and space parameters of the laser light source, an intuitive time and space function of the excitation source can be plotted, as shown in Figure 2.

**Figure 3:**
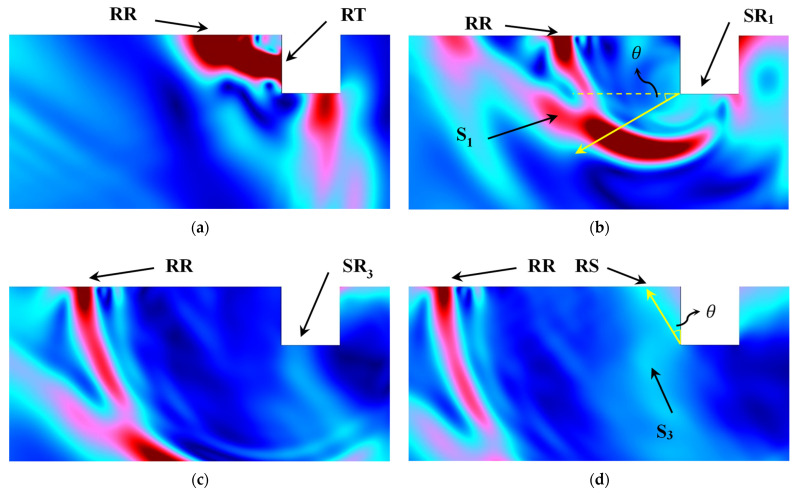
The acoustic field around the notch at (**a**) 3.81 μs; (**b**) 4.21 μs; (**c**) 4.61 μs; (**d**) 4.81 μs.

A rectangular model the size of 30 mm × 10 mm is built. The two kinds of material used in this model are aluminum and steel. Due to the two-dimension model involved in this work, the incident light source is described in the space–time domain, where the radius of the light source is 100 μm and the rise time of the laser pulse is 15 ns. The total simulation time duration is 10 μs with time steps of 10 ns. A free triangle mesh is used with a spatial step of 10 μm for areas near defects, and 100 μm for the rest of the area. In order to meet the precision requirements of elastic wave propagation, the spatial step has to be controlled below one quarter of the minimum wave wavelength. The minimum wave wavelength is calculated as 226 μm by the maximum frequency of 13.24 MHz, which is obtained by the equation of (√2·vr)/(pi·a0), where a0 is the radius of laser set as 100 μm and vr is the velocity of the Rayleigh wave. So, the time step and spatial step are suitable for the simulation requirements in this work. Meanwhile, CFL is calculated by the time step and the spatial step is 0.01 less than 0.02 meeting the simulation convergence. The size of the surface notches is set as three types of notches according to the definition in Section 2.1, and the arguments of simulation results on the base of their types are in the next section. To observe the path of acoustic waves at the crack, Figure 3a–d represent the acoustic field at surface defects at 3.81 μs, 4.21 μs, 4.61 μs, and 4.81 μs of the simulation process, respectively.

The simulation results of the defect are set sized with a length × width of 0.8 mm × 0.8 mm with the size ratio of w/d equal to 1 belonging to the narrow defect type according to the definition of defect types in Section 2.1; these are presented in Figure 3. So, the waves’ path of reflection, transmission, and scattering waves around the surface notch in Figure 1 should be observed by the FEM. Four transient moments in the process of wave propagation are picked corresponds to waves’ path presented in Figure 1. Figure 3a shows that incident surface wave R meets the left face of defect, and one part of it forms the wave RT propagating along the surface, the other part of it forms the reflected wave RR in the opposite direction of the incident wave. At the other moments, the reflected surface wave RR propagates along the surface and reaches the location in Figure 3d. This procedure is path ① in Figure 1. The surface wave RT continues to propagate along the bottom of the defect and arrives at the defect sidewall, and the first modal conversion occurs at the M point resulting in the surface wave SR1 and shear wave components S_1_ being generated, as shown in Figure 3b. It is seen that the surface wave SR_1_ propagates along the bottom MN, and the shear wave S_1_ propagates into the material. Consistent with the analysis of the energy of the scattered waveform in Section 2.1, the scattering angle of wave S_1_ is 30° labeled with a yellow line, which can be determined by detecting the maximum amplitude of the scattering waves at the tip of crack. The scattering angle of wave S_1_ leads to this wave propagating into the material and not being detected at the specimen surface. It illustrates the correctness of the theoretical analysis of Formula (1), and also shows that the subsequent generation of surface wave components RS is not by the surface wave RT, but in other processes. Surface wave SR_1_ undergoes the second modal transition at the point N generating the reflected surface wave SR_3_ shown in Figure 3c. The Rayleigh wave SR_3_ is divided into three parts, which are the surface wave SR_2_ along the surface of the right face of the notch, the shear wave S_2_ scattering into the material, and the reflected Rayleigh wave SR_3_ along the bottom. The Rayleigh wave SR_3_ continues to propagate and forms the transmitted Rayleigh wave RT. This is wave path ② in Figure 1. The surface wave SR_3_ propagates along the bottom MN of the notch, and when it meets the tip M, a third modal transition occurs and the shear component S_3_ is generated at the tip M in the material shown in Figure 3d. This is path ③ in Figure 1. The scattering angle of wave S_1_ also is 30° in clockwise rotation along the direction of wave propagation giving rise to that it spreads towards the specimen surface. The directivity of scattering wave S_1_ produced at the M-spot is labeled using the red line. Its scattering angle also is measured as 30° by the manual manipulation. The value of the scattering angle accords with that in Formula (2). Comparing Figure 3b,d, it can be seen that the surface wave RS is not generated until the shear wave S_3_ reaches the specimen surface. And shear wave S_3_ is scattered by the third mode conversion in the path ③ at the tip M of the notch. Therefore, it can be proved that the surface wave RS is generated by the shear wave S_3_ not by the surface wave RT. The theoretical analysis in Section 2.1 is verified with the simulation results that are obtained using the finite element method. 

Because the surface notch in Figure 3 are of the narrow defect type, the defect depth measurement theory of Formula (2) can be applied to detect the defect depth. The simulation results of the FEM show the processes of wave generation, propagation, and modal conversion that occurs at the defect, and demonstrate the correctness of defect depth measurement theory proposed in Section 2.1. It shows that defect depth can be evaluated by the difference between the surface wave RR and RS arrival time. In order to obtain further analyses of the measurement theory proposed in Section 2.1, it is necessary to design simulation models for different defect models of extremely narrow defects and wide defects with the different size ratios to test their measurement applicability.

### 2.3. Simulation Results for Narrow and Extremely Narrow Defects

Two materials of aluminum and steel were used in the simulation model for laser ultrasonic simulation by the FEM in this work. This section is for the results of the narrow defects and extremely defects. The property parameters of aluminum and steel are in Table 2.

According to Table 2, the value of β in Equation (2) is 0.6573 in steel and 0.6578 in aluminum. Narrow defects and extremely narrow defects are set on the aluminum and steel model.

**Figure 4:**
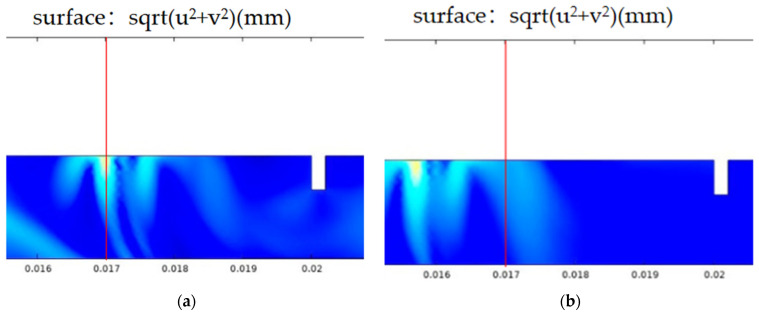
Surface wave sound field diagram: (**a**) 4.71 μs; (**b**) 5.18 μs.

**Figure 5:**
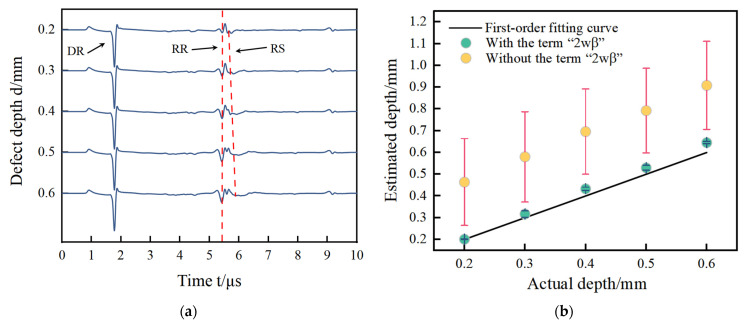

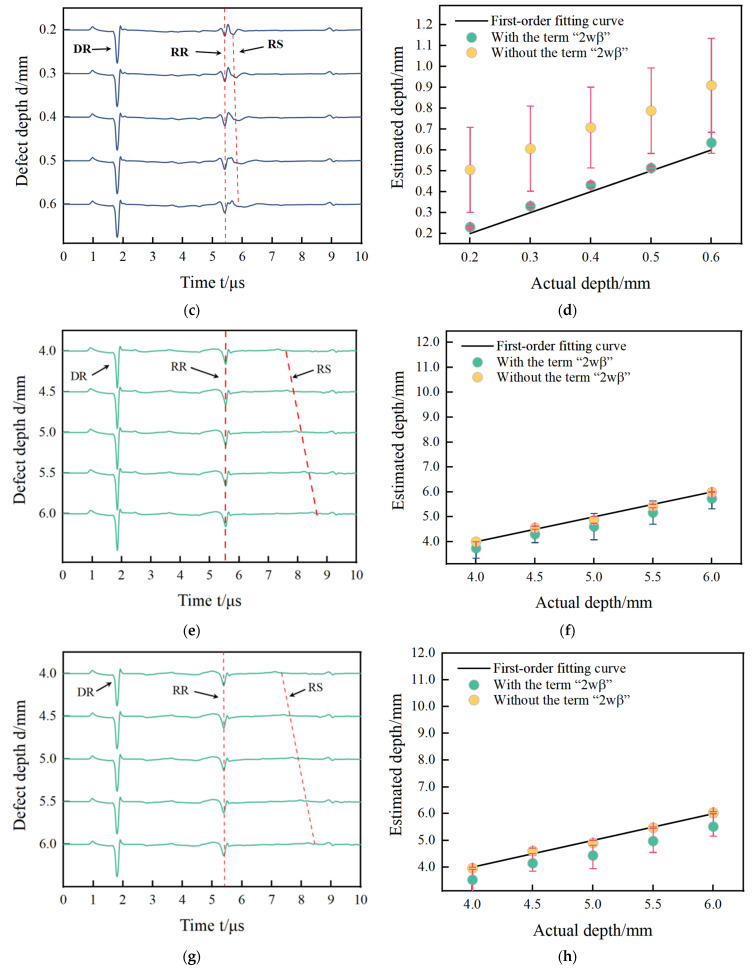
The results in (**a**–**d**) are for narrow defects, where (**a**) for acoustic signals and (**b**) for estimated results with and without the corrected term are aluminum, (**c**) for acoustic signal and (**d**) for estimated results with and without the corrected term for steel. The results in (**e**–**h**) are for extremely narrow defects, where (**e**) represents acoustic signals and (**f**) represents the estimated results with and without the corrected term for aluminum, (**g**) for acoustic signals, and (**h**) for estimated results with and without the corrected term for steel.

Some waveform peaks are smaller, and under the defect of 0.5 mm deep and 0.2 mm wide in Figure 4, the surface wave RS attenuates significantly during propagation, resulting in a smaller peak value. Here, the sound field diagram from the defect simulation model is combined to distinguish the arrival time of the surface wave RS. The position of the probe set in the model is fixed. As long as the surface wave RS is observed and reaches the fixed position, the corresponding time of the sound field graph at this point can be recorded to obtain the accurate arrival time of the surface wave RS. In Figure 4, (a) is the sound field graph at time 4.71μs, and (b) is the sound field graph at time 5.18μs. It can be seen that the propagation path of the surface wave RS is more obvious in the sound field diagram, and the arrival time of RS can be determined by combining the probe position. The probe values are all at 0.017 m. Observing the sound field graph, the moment when the waveform peak reaches this point is the arrival time of the surface wave RR and RS. Figure 5 shows the arrival time of the RS waves obtained using this method.

The simulation signals acquired by the FEM for two kinds of defects are in shown Figure 5a,c,e,g. The sizes of defects in Figure 5a,c are the same, the widths of the defects are all 0.2 mm, and the depth of the defects are 0.2 mm, 0.3 mm, 0.4 mm, 0.5 mm, and 0.6 mm. The sizes of the defects in Figure 5e,g are also the same, the widths of defects are all 0.2 mm, and the depth of defects are 4.0 mm, 4.5 mm, 5.0 mm, 5.5 mm, and 6.0 mm. Their size ratios satisfy the requirements defined in Section 2.1. The defects’ size is shown in Figure 5a,c defects with size ratio from 0.3 to 1 are narrow defects. Figure 5e,g defects with a size ratio from 0.03 to 0.05 are extremely narrow defects. The size design of the defects is consistent with the experimental specimens in the following section. The arrival times of the direct wave, reflected wave, and scattering wave named DR, RR, and RS are shown in Table 3. The estimated results with and without the item “2wβ” are shown in Figure 5b,d,f,h, corresponding to Figure 5a,c,e,g, respectively. The estimated values and their errors are presented in Table 3.

In Figure 5a,c,e,g, the waves DR, RR, and RS are acquired at the narrow defect and extremely narrow defect. The wave DR is the direct Rayleigh wave generated by the laser line source. The wave RR corresponding to the wave RR at path ① is a reflected wave at the defect. The wave RS corresponding to the wave RS at path ③ is a mode-converted wave on arrival on the surface of specimen. In Figure 5, the distance between the excitation points and the reception point is the same resulting in that the reception time of the waves RR are the same time measurement of 1.78 μs for all defects. The distance from the reception point to all defects is almost fixed. For narrow defects, the arrival time of waves RR is 5.41 μs for both aluminum and steel. For extremely narrow defects, the arrival times of waves RR are 5.54 μs for aluminum and 5.41 μs for steel. For the arrival time of wave RS, this is related to the defect depth. So, the time delay of the waves RS is observed in Figure 4, and they are picked to estimate the notch depth using Equation (2). To compare the measurement results with and without the corrected item “2wβ”, the estimated depths are shown in Figure 5b,d,f,h for narrow defects and extremely narrow defects. 

To show the effect of the corrected item more clearly on the evaluated depth, two times of the scale of the *y*-axis than that of the *x*-axis are both shown in Figure 5b,d,f,h. Two phenomena are observed in Figure 5a,c,e,g. One is that the measured depth and actual depth are close to a linear relationship. The other phenomenon is that the estimated results with the corrected item are lower “2wβ” than those without the corrected item. The reasons for these phenomena can be found in another form of Equation (1) as follows:

(7) y=kx−b

where y is the estimated depth d of the defect; x is the time difference of Δt in Equation (2); b is the corrected item “2wβ”; and k is the coefficient of $\frac{\cos\theta v_{r}v_{s}}{\left(\cos\theta -\sin\theta \right)v_{s}+v_{r}}$, which is the constant value due to the fact that the property parameters of the detected material are known. The time difference and the estimated depth are a linear relationship and the corrected item “2wβ” does not change this relationship, as shown in the negative exit before the item “b” in Equation (7). So, the difference between the estimated results with the corrected item and the estimated results with the corrected item is just equal to twice of the defect width as “2wβ”. To obtain a clearer comparison between the narrow defect and the extremely narrow defect, the relative errors for two types of defects are presented in Table 3.

For the No. 1~10 defects, the relative errors using the corrected item “2wβ” less than 16% are much smaller than that without the corrected item “2wβ” more than 51.38%. So, for the depth measurement of narrow defects for both aluminum and steel, the corrected item “2wβ” cannot be omitted in Equation (2). For No. 11~20 defects, the relative errors using the corrected item “2wβ” less than 11.88% are a little more than that without the corrected item “2wβ” less than 2.60%. The item “2wβ” in Equation (2) has few corrected effects on the measured depth. So, for the depth measurement of extremely narrow defects, the corrected item “2wβ” can be omitted in Equation (2). All in all, these conclusions are consistent with the theoretical analysis in Section 2.1, where the corrected item “2wβ” cannot be omitted for the depth measurement of narrow defects and can be omitted for the depth measurement of extremely narrow defects.

### 2.4. Simulation Results for Wide Defect

**Figure 6:**
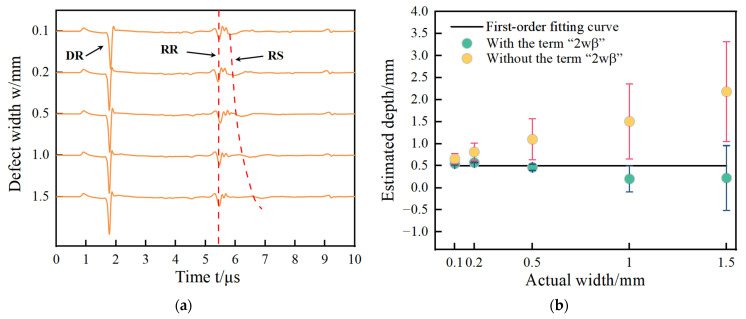
(**a**) Acoustic signals at wide defects; (**b**) estimated results with and without the corrected term for wide defects.

Narrow and extremely narrow defects were analyzed in Section 2.3. In this section, wide defects are studied. Five notches under the definition of wide defects are set out on the aluminum model and their sizes are shown in Figure 6 and Table 3. The No. 1~5 defects with a size ratio of 2 are wide defects. The simulation signals acquired by the FEM are shown in Figure 6a. The arrival times of the direct wave DR, reflected wave RR, and scattering wave RS are shown in Table 3. The estimated results with and without the term “2wβ” are shown in Figure 6b. The estimated values and their errors are presented in Table 4.

The arrival time of the RS waves at the wide defect are extracted in Figure 6a and the estimated depths of notches using Equation (2) with and without the corrected item “2wβ” are shown in Figure 6b. It can be seen that the linear relationship between the estimated depth and actual depth are just like that of the narrow defect in the aluminum model in Figure 5b and that of the steel model in Figure 5d disappears. The corrected item “2wβ” has an insignificant influence on modifying estimated depths to obtain more exact results. According to the ultrasonic propagation process in Figure 1 in Section 2, the propagation processes and arrival time of surface waves RR and RS are always affected by the defect’s size, but the generation process of the two surface waves are very different, which directly influence the estimated results of the defect depth measurement. Depth and width have different effects on the estimated results. The surface wave RR is generated by the direct reflection at the interface on the left side of the defect. During the procedure from its generation to the reception, the sound path of the surface wave RR is not involved in the defect width. The surface wave RS is generated at path ③ in Figure 1, and this sound path includes both depth and width. So, the change in defect size will have a significant impact on the arrival time of the wave RS. The sound path to generate wave S3 includes the crack depth due to wave RT propagation and two times of defect width due to the propagation of waves SR1 and SR3. It is presented in Figure 1 and verified in Figure 2. For narrow defects and extremely narrow defects, the defect width is relatively small, and the sound path and time interval between the three modal transitions are also small, so the impact on the surface wave RS is also small. When using Equation (2) to calculate the defect depth, accurate depth detection can be obtained using the width correction item or directly ignoring the effect of width. For wide defects, the defect width is relatively large, and an excessively long sound path will attenuate the amplitudes of wave SR1 and SR3. So, the arrival time of the surface wave RS becomes inaccurate.

In order to examine the influence of the defect width more accurately, the defect depth is set to a constant value as d = 0.5 mm. By changing the form of Equation (2), the relationship between time delay Δt and the size of defect can be described as follows:

(8) $$\Delta t=\frac{\left(\cos\theta -\sin\theta \right)v_{s}+v_{r}}{\cos\theta v_{r}v_{s}}(d+2w\beta )$$

**Figure 7:**
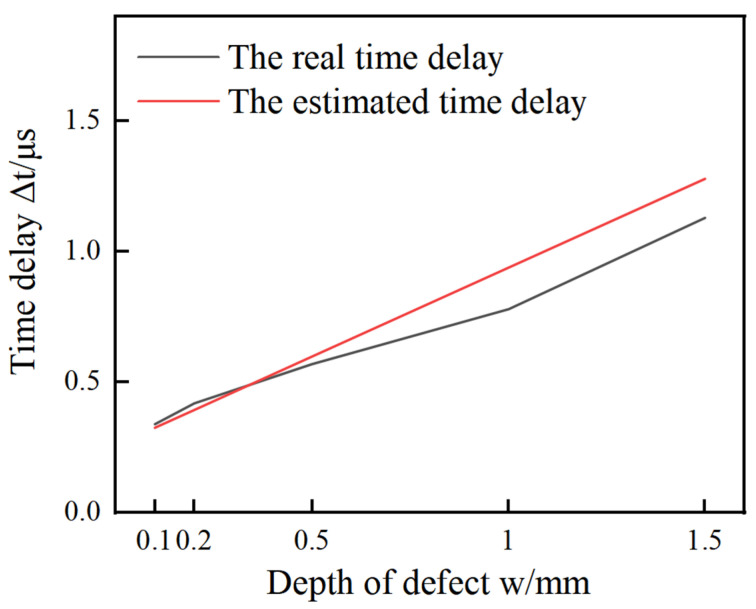
Time delay versus depth of defect.

Accordingly, as shown in Equation (8), the function Δt is relative to the defect width and depth. As the defect depth is a constant, the function Δt is a monotonic function only related to the defect width. And then the theoretical time difference corresponding to different defect widths is obtained in Figure 7. Table 5 provides the theoretical values and measurement values by the FEM of the time difference corresponding to different widths under the same defect depth.

In Table 5, the depth of defects on the five specimens are the same at 0.5 mm, but the width of them is at 0.1 mm, 0.2 mm, 0.5 mm, 1.0 mm, and 1.5 mm. The front three specimens belong to the narrow defects category, the back two specimens belong to wide defects. The time delay extracted from defects Nos. 1, 2, and 3 is basically consistent with the theoretical time difference, and the relative error of the calculated defect depth is also small. The data change from No. 4. The time delay extracted from these three groups of defects is quite different from the theoretical time delay, and the relative error of the calculated defect depth is also very large. Therefore, Equation (2) cannot be used for width defect depth measurement.

### 2.5. The Up and Down Bound of Narrow Defect Size Ratio Analysis

**Figure 8:**
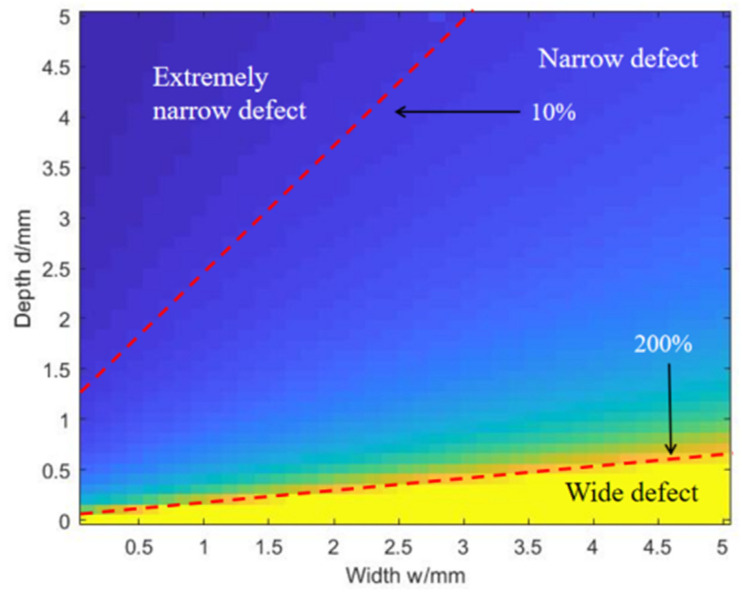
The relative errors in the plane of width vs. depth in the image.

This section covers how to decide the up and down boundaries of the narrow defect size ratio. So, 2050 specimen models including all three types of the surface notches, whose depth ranges from 0.1 mm to 5.0 mm with the interval of 0.1 mm, and width ranges from 0.1 mm to 0.5 mm with the interval of 0.01 mm, are set on models in the FEM. The evaluated depths are obtained using Equation (2) without the corrected item “2wβ”. Their relative errors are presented in Figure 8.

In the actual measurement, the term “2wβ” will cause the relative error, which is “2wβ/d”, in the results, but the influence of this deviation is inconsistent for different defects. The relative errors caused by the term “2wβ” in different defect measurements are given in Figure 8. In the case of the same defect depth, which is the same row of data in the figure, the influence of term “2wβ” on the results continues to increase with the increase in width, and all rows show the same rule. This phenomenon shows that under the condition of a certain defect depth, the influence of term “2wβ” on the measurement results continues to increase with the increase in the width. In the case of the same defect width, which is the same column of data in the figure, the opposite of the effect of width occurs, as the influence of term “2wβ” on the results is decreasing with the increase in depth, and all columns show the same rule. This shows that under the condition of a certain defect width, the influence of term “2wβ” on the measurement results decreases continuously with the increase in depth. It can be seen from the figure that when the error falls below 10% and rises above 200%, the area connected by these defects constitutes a clear dividing line, and the measurement range of narrow defects can be delineated accordingly.

## 3. Experimental Scheme and Results Analysis

### 3.1. Experimental Setup and Specimens

**Figure 9:**
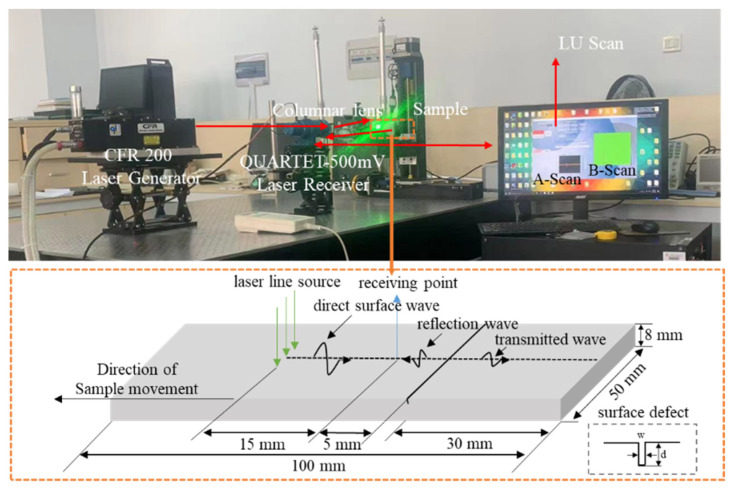
Laser ultrasound experimental platform and specimen.

This paper uses the thermal elastic excitation principle and line source focusing method to build a laser ultrasonic detection platform, which mainly includes two parts: a hardware system and a software system, as shown in Figure 9. The laser ultrasonic testing platform uses the CFR200 laser generator as the laser excitation part, and the emitted laser wavelength is 1064 nm, pulse width is 11 ns, and pulse repetition rate is 20 Hz. The QUARTET-500 mV laser ultrasonic receiver based on the principles of the Michelson interferometer is used as the reception part, the detection sensitivity is 1 × 10^–5^ nm/Hz^½^, the laser wavelength is 532 nm, and the bandwidth is 100 kHz to 20 MHz. The laser emitted by the excitation part is excited by a cylindrical lens with a focal length of 200 mm on the sample surface to form a linear source to excite the surface acoustic wave, and the echo signal is received by the laser interference section. The laser ultrasonic testing platform is equipped with an automatic scanning frame, which can complete the A-Scan acoustic signal reception and B-Scan imaging of the test sample. Using fixed excitation points and receive points, and employing Lu Scan software 2015 v2.3.0, a scanning scheme is set to control the automatic scanning rack for placing samples and to achieve a horizontal scan with a step of 0.12 mm and a scanning distance of 30 mm. During the initial scan, the distance between the excitation points and the receiving point is 15 mm, and the distance between the receiving point and the defect is 5 mm, as shown in Figure 9. On the basis of the surface notch size definition in Section 2.1, the wide, narrow, and extremely narrow defects are cut on the aluminum and steel specimens. The dimensions of the surface notches on the specimens are shown in Table 6.

**Figure 10:**
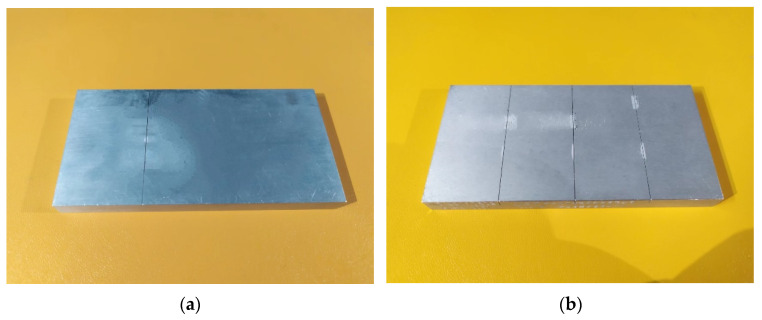
(**a**) One defect on the surface of specimen; (**b**) three defects on the surface of specimens.

It can be seen that twelve aluminum specimens and six steel specimens are used in this work. For the aluminum specimens, the No. 1–No. 5 specimens are narrow defects with a crack size ratio ranging from 0.3 to 1; the No. 6–No. 10 specimens are extremely narrow defects with a crack size ratio ranging from 0.03 to 0.05; and the No. 11–No. 12 specimens are wide defects with a crack size ratio equal to 2. For the steel specimens, the No. 1–No. 3 specimens are narrow defects with a crack size from 0.5 to 1, No. 4–No. 6 specimens are extremely narrow defects ranging from 0.05 to 0.03. To save the material of the specimen, two types of size of specimen were designed in this work. One type is one defect on the surface of the specimen, and the other type is three defects on the specimen’s surface. They are shown in Figure 10.

### 3.2. Experimental Results Analysis

Two materials of specimens with three types of defects are performed on experimental platform built in Section 3.1. The acquired acoustic signals, the estimated results, and their analysis are presented as follows.

#### 3.2.1. The Experimental Results for Narrow and Extremely Narrow Defects

**Figure 11:**
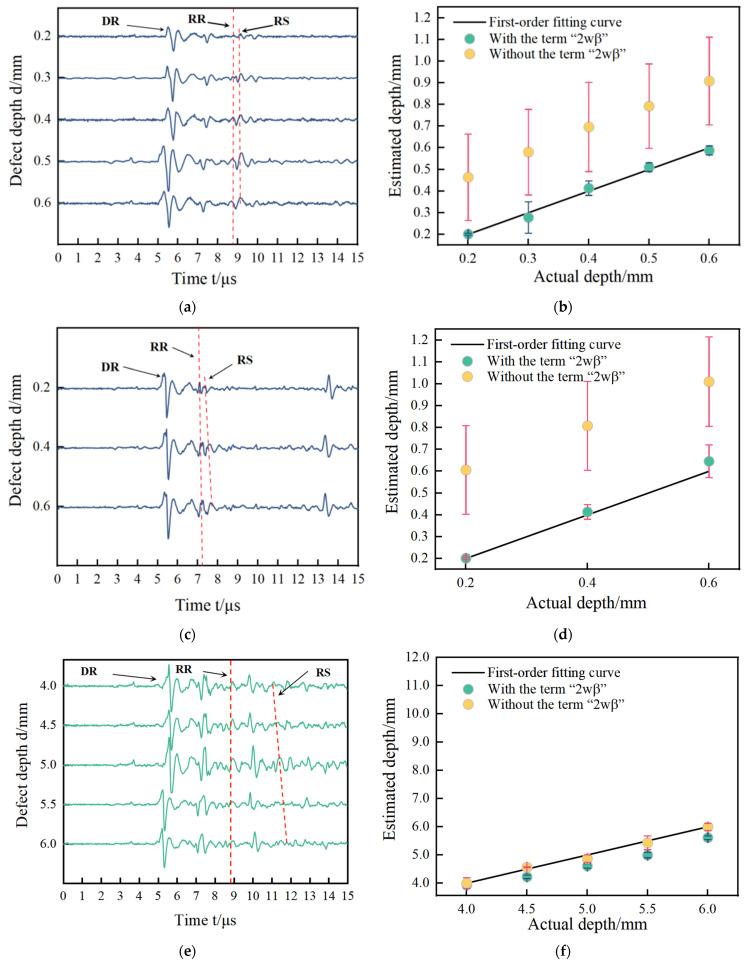

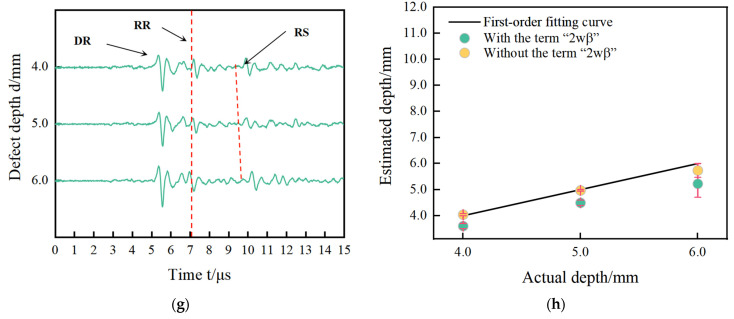
The experimental results in (**a**–**d**) are for narrow defects, where (**a**) represents acoustic signals and (**b**) represents estimated results with and without the corrected term are aluminum, (**c**) for acoustic signals, and (**d**) for the results of steel. The experimental results in (**e**–**h**) are for extremely narrow defects, where (**e**) represents acoustic signals and (**f**) represents estimated results with and without corrected term for aluminum, (**g**) for acoustic signal, and (**h**) for the results for steel.

The acoustic signals in the time domain for the No. 1–No. 5 aluminum specimens and No. 1–No. 3 steel specimens with narrow defects are presented in Figure 11a,c, and the estimated results with and without the corrected item “2wβ” are presented in Figure 11b,d. The acoustic signals in the time domain for the No. 6–No. 10 specimens and No. 4–No. 6 steel specimens with extremely narrow defects are presented in Figure 11e,g, and the estimated results are in Figure 11f,h.

**Figure 12:**
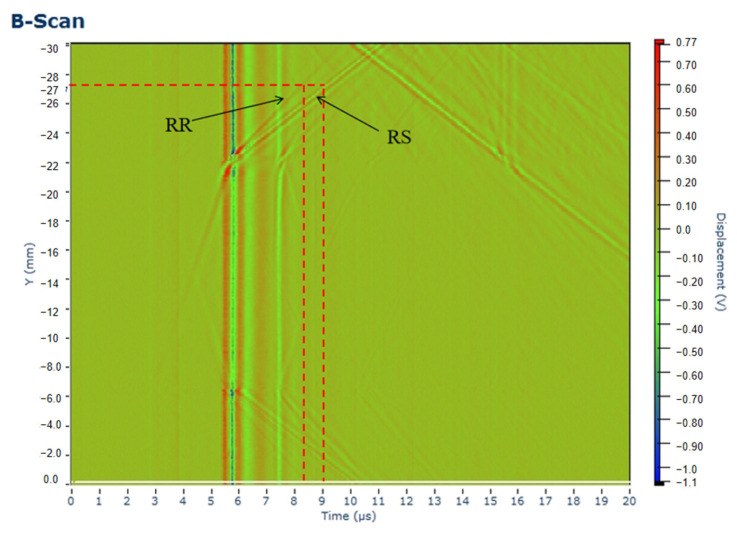
B-scan image of aluminum alloy with a depth of 0.2 mm.

During the scanning process, the distance between the excitation point and receiving point is fixed at 15 mm, and the theoretical arrival time of square wave DR appears at around 5 μs, and the waveform corresponding to t = 5.5 μs in Figure 11a is the direct surface acoustic wave signal excited by the laser. In addition, when the distance between the receiving point and the defect is 5 mm, combining with a surface wave velocity of 2940 m/s, the first peak of the defect reflection echo can be calculated. That is, the surface wave RR roughly appears at 5.0 + 3.4 = 8.4 μs. Theoretical calculation shows that the received arrival time of surface wave RR is 8.4 μs. According to the scattering phenomenon of the defect tip waveform proposed in Section 2.1, there are two peak values of surface wave RR and RS in the defect reflection echo. The time at which surface wave RR occurs has been determined, and the first larger peak value after its appearance is the surface wave RS. According to the calculation results and the tip waveform scattering theory, the arrival times of surface waves RR and RS in the direct surface wave and the reflected echo at the defect are consistent with the arrival times of the two wave forms detected in the experiment, which prove that the experimental results are good. In order to locate the time when the waveform appears, B-scan images are combined here to distinguish the arrival times of surface waves RR and RS accurately and clearly. The B-scan image of the defect at a depth of 0.2 mm is shown in Figure 12, corresponding to the 0.2 mm acoustic wave image in Figure 11a. The two diagonal lines in Figure 12 represent RR and RS, respectively. The detection point is located on the left side of the defect at −22 mm on the vertical axis, and is 5 mm away from the defect. Therefore, the arrival time of RR at −27 mm on the vertical axis is 8.4 μs, and the arrival time of RS is 9.0 μs. In the experiment, the arrival time of the RR wave and RS wave can be determined using time-domain positioning and B-scan images.

It is noteworthy that in Figure 11c, in addition to observing the wave DR and wave RR, the reflected waves, which are from the other defects and which arrive between DR and RR, are also received. Because the No. 6–No. 10 specimens belong to the second type of specimen shown in Figure 11b, in Figure 11b,d,f,h, the first order fitting curve of the actual depth versus the measured depth with and without the corrected item “2wβ” are also shown. The linear relationships between the actual depth and measured depth are observed in Figure 11, and the estimated results without the corrected item are lower close to “2wβ” than the estimated results with the corrected item shown in Figure 11. These phenomena are in agreement with the simulation results in Section 2.2, where the explanation for them is also given. In Figure 11f,h, the estimated results with corrected item “2wβ” are very close to the estimated results without corrected item “2wβ”. These results for extremely narrow defects on No. 6–No. 10 specimens are consistent with the simulation results in Section 2.3. For extremely narrow defect depth measurement, the corrected item “2wβ” has limited effects on the evaluated depth of defect so that it can be ignored during the measurement process. The arrival times of the waves DR and RR, and the relative errors with and without the corrected item “2wβ”, are presented in Table 7.

For narrow defects, the maximal relative error with corrected item “2wβ” is less than 8%, and the minimum relative error without corrected item “2wβ” is more than 41%. So, the corrected item “2wβ” plays a very important role in obtaining the accurate depth measurement results for narrow defects. However, for extremely narrow defects, the relative errors with and without the corrected item “2wβ” are both less than 13%. Because the surface defect width is so small that the estimated results regarding width have a worse effect, the relative errors with corrected item are bigger than those without the corrected item. Therefore, for the depth measurement of extremely narrow defects, the corrected item “2wβ” from the defect width in Equation (2) is ignored. Based on the experimental results, the simulation results and theoretical analysis are validated very well for aluminum specimens. 

#### 3.2.2. The Experimental Results for Wide Defects

**Figure 13:**
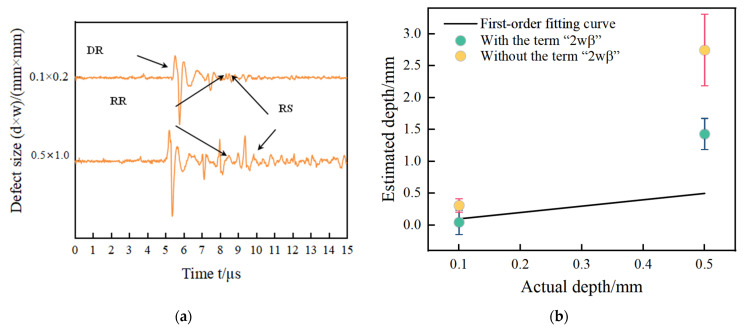
Aluminum experimental data of (**a**) wide defect (**b**) the estimated results with and without the term “2wβ”.

The acoustic signals in the time domain for No. 11 and No. 12 specimens with wide defects are presented in Figure 13a and the estimated results with and without the corrected item “2wβ” are presented in Figure 13b and Table 8.

For wide defects, the relative errors with and without corrected item “2wβ” are both more than 53%, leading to the failure of defect depth measurement using the mode conversion method based on laser ultrasonics. Meanwhile, the linear relationship between the actual depth and estimated depth is gone due to the measured method based on Equation (1) not working anymore. It can be seen that the experimental results for wide defects are consistent with the simulation results on the steel specimens in Section 2.3. The experimental results for wide defects are the same as the phenomena observed in Figure 6a which demonstrates that the calculation method based on Equation (1) proposed in this work can be applied for wide defects’ depth measurement.

### 3.3. The Comparison with Cooper’s and Jeong’s Results

The view in Cooper’s [18] and Jeong’s studies [24] is that the surface wave RT excites the shear wave S3 when it first reaches the M point, so the formula provided in the articles does not mention the effect of the width of the defect, i.e., ignores the 2wβ term in Equation (2). However, when measuring defect depth, the mathematical correction in [18] is made according to experimental data, and a formula considering defect width, which is Equation (2), is given. However, the principle for mathematical correction is not explained. However, in [24], the depth measurement errors of defects generated by the negligence of the width of defect are overlooked, because the first-order fitting curve of actual depth versus measurement depth is applied to estimate defect depth and no attention is paid to the effect of the defect width on the depth evaluation of the defect. The detected defects studied in [18] all belong to the narrow defects with the same width at 0.1 mm, satisfying the definition of narrow defects in Section 2.1. According to Section 2.1, in [24], the front two defects are narrow defects, and the back four defects are extremely narrow defects. So, different from [18], two types of defect are involved in [24]. It can be seen in Table 8 that the relative errors of measured defect depth by ignoring corrected term “2wβ” range in 17.89%~131.91%, which is a very considerable value leading to the failure of depth measurement. Considering the corrected term “2wβ”, the relative errors are significantly reduced ranging in 1.47%~11.31%. and the measured results are very close to the true value. 

After calculation, ignore the correction term “2wβ”. The relative error of the measured defect depth is between 17.89% and 131.91%, which is a very significant value, leading to the failure of the depth measurement. Considering the correction term “2wβ”, the relative error is within 11.31%, and the measurement results are very close to the true values. On the other hand, the calculated surface mode-converted wave RS is generated by two reflections of wave S3 along the bottom of the defect. Therefore, for measuring the depth of narrow defects, the correction term “2wβ” cannot be ignored [18]. However, Jeong only studied narrow defects in [24]. According to the theoretical analysis in Section 2.1, for extremely narrow depth measurements, correct the item “2wβ”. It has almost no impact on the estimation results. The same conclusion was obtained through the finite element simulation results in Section 2 and the experimental data in Section 3. For narrow defects, the relative errors without correction terms are 48.16% and 17.89%, which are much greater than the errors with correction terms. For extremely narrow defects, the relative error without a correction term is close to that with a correction term. It can be seen that Jeong’s experimental data also confirm the measurement methods for narrow defects and extremely narrow defects.

Compare with and without correction item “2wβ” in [18]. The estimated depth difference between the two is almost equal to 1.3 times the width of the defect. Due to the narrow defect detected in [18], Cooper observed that the correction term “2wβ” cannot be omitted. The phenomenon was verified through this work. However, the detection defects in [24] include narrow defects and extremely narrow defects. A comparison with [18] with and without correction item “2wβ” provides that the estimation depth difference between them is significantly different, and the extremely narrow defect estimation results in [24] are very close. For narrow defects, the revised project “2wβ” cannot be ignored in depth measurement. For extremely narrow defect estimation results, with and without correction terms “2wβ”, the estimated depths are close to each other. All in all, the mode conversion method based on the laser ultrasonics proposed in this work is theoretically presented using an analytic formula based on the paths of the RS and RR’s waves. It is verified by the simulation results by the FEM and the experimental data by the total optic platform. In addition, this measurement method is also applicable for Cooper’s [18] and Jeong’s studies [24].

## 4. Discussion

Through the analysis of wave paths at the surface notches, the mode conversion method based on laser ultrasonics is proposed in this work. The time difference in the reception of wave RS and wave RR related to the crack size is extracted to measure the defect depth. The FEM and experimental method are both performed to validate this depth measurement method. These conclusions are as follows:

(1) The defect width effects the precision of defect depth measurement using the time characteristic of scattering acoustic wave propagation to size defect depth. Three types of defects are defined in this work according to the width and depth ratio of the defect. The main difference in their measurement method is how to deal with the corrected item “2wβ”. For narrow defects, the term “2wβ” cannot be omitted. For the extremely narrow defects, the term “2wβ” can be omitted. However, for wide defects, the mode conversion method does not work anymore.
(2) The acoustic fields including reflected, scattered, and transmitted fields at the defects are observed. That scattering wave RS at the defect tip is generated by wave S_3_ after two reflections at the bottom of defect is confirmed. The scattering angle of scattering wave S_3_ at the defect tip is measured as 30°. These two key parameters in Equation (2) are determined by the FEM. Moreover, aluminum and steel models with three types of defects are built. The simulation results are consistent with the theoretical analysis.
(3) The non-contact experimental platform with the auto scanning setup is built. The depths of eighteen defects covering three types of defects made on the surface of aluminum and steel specimens are measured. The experimental results in Section 3 are consistent with the simulation results by the FEM in Section 2 and verify the theoretical analysis based on Equation (2).
(4) Moreover, the results in [18,24] are redisplayed using the FEM. The further explanation for their results is presented. After that, detected defects are classified and the measurement methods for narrow and extremely narrow defects are applied, the conclusions in [18,24] are demonstrated as being the same as in this work.

For a long time, defect depth evaluation using laser ultrasonics has been widely seen as a single parameter inversion problem in the frequency and time methods. Actually, the defect width significantly effects the defect depth measurement. By defining the defect size ratio, two parameters involved in the inversion problem are shown using Equation (2). The simulation results and experimental data validate this mode conversion method based on laser ultrasonics in this work.

## Figures and Tables

**Table 1:** Time and space parameters of laser source.

Pulse Rise Time/s	Pulse Duration/s	Gaussian Beam Radius/m
5 × 10^−9^	3.1 × 10^−6^	1 × 10^−4^

**Table 2:** The simulation parameters of aluminum and steel.

Parameters	Values of Aluminum	Values of Steel
Longitudinal wave	6300 m/s	5900 m/s
Transverse wave	3090 m/s	3230 m/s
Rayleigh wave velocity	2940 m/s	3070 m/s
Heat conductivity coefficient	238 (W/(m·K))	45 (W/(m·K))
Thermal expansion coefficient	23 × 10^−6^ 1/K	10.8 × 10^−6^ 1/K

**Table 3:** Defect size and estimated results.

No.	Type of Defect	d /mm	w /mm	RR /μs	RS /μs	Δt/μs	Estimated Depth with Corrected Item	Relative Errors	Estimated Depth without Corrected Item	Relative Errors
1	Aluminum narrow in Figure 5a,b	0.2	0.2	5.41	5.65	0.24	0.2009	0.44%	0.4638	131.91%
2		0.3	0.2	5.41	5.71	0.30	0.3168	5.61%	0.5789	93.26%
3		0.4	0.2	5.41	5.77	0.36	0.4328	8.20%	0.6957	73.93%
4		0.5	0.2	5.41	5.82	0.41	0.5294	5.88%	0.7924	58.47%
5		0.6	0.2	5.41	5.88	0.47	0.6454	7.56%	0.9083	51.38%
6	Steel narrow in Figure 5c,d	0.2	0.2	5.41	5.66	0.25	0.2301	15.06%	0.5049	152.44%
7		0.3	0.2	5.41	5.72	0.31	0.3311	10.36%	0.6059	101.95%
8		0.4	0.2	5.41	5.76	0.35	0.4321	8.02%	0.7068	76.71%
9		0.5	0.2	5.41	5.80	0.39	0.5129	2.57%	0.7876	57.52%
10		0.6	0.2	5.41	5.86	0.45	0.6340	5.67%	0.9088	51.47%
11	Aluminum extremely narrow in Figure 5e,f	4.0	0.2	5.54	7.61	2.07	3.7375	6.56%	4.0004	0.01%
12		4.5	0.2	5.54	7.90	2.36	4.2979	4.49%	4.5608	1.35%
13		5.0	0.2	5.54	8.06	2.52	4.6071	7.86%	4.8701	2.60%
14		5.5	0.2	5.54	8.35	2.81	5.1676	6.04%	5.4305	1.26%
15		6.0	0.2	5.54	8.64	3.10	5.7280	4.53%	5.9909	0.15%
16	Steel extremely narrow in Figure 5g,h	4.0	0.2	5.41	7.37	1.96	3.5249	11.88%	3.9583	1.04%
17		4.5	0.2	5.41	7.69	2.28	4.1433	7.93%	4.6045	2.32%
18		5.0	0.2	5.41	7.84	2.43	4.4332	11.34%	4.9075	1.85%
19		5.5	0.2	5.41	8.12	2.71	4.9743	9.56%	5.4729	0.49%
20		6.0	0.2	5.41	8.40	2.99	5.5154	8.08%	6.0384	0.64%

**Table 4:** Defect size and estimated results of wide defects.

No.	d/mm	w/mm	RR/μs	RS/μs	Δt/μs	Estimated Depth with Corrected Item	Relative Errors	Estimated Depth without Corrected Item	Relative Errors
1	0.5	0.1	5.46	5.80	0.34	0.5493	9.85%	0.6571	31.41%
2	0.5	0.2	5.46	5.88	0.42	0.5734	14.69%	0.8117	62.34%
3	0.5	0.5	5.49	6.06	0.57	0.4642	7.16%	1.1016	120.31%
4	0.5	1.0	5.46	6.24	0.78	0.2014	59.72%	1.5074	201.48%
5	0.5	1.5	5.46	6.59	1.13	0.2213	55.74%	2.1838	336.76%

**Table 5:** Defect width and time delay.

Defect Width/mm	Theoretical Δt	Actual Δt
0.1	0.33	0.34
0.2	0.40	0.42
0.5	0.60	0.57
1	0.94	0.78
1.5	1.28	1.13

**Table 6:** The specimen surface defects size.

No.	Depth d/mm	Width w/mm	Defect Type	Material
1	0.2	0.2	Narrow	Aluminum
2	0.3	0.2	Narrow	
3	0.4	0.2	Narrow	
4	0.5	0.2	Narrow	
5	0.6	0.2	Narrow	
6	4.0	0.2	Extremely narrow	
7	4.5	0.2	Extremely narrow	
8	5.0	0.2	Extremely narrow	
9	5.5	0.2	Extremely narrow	
10	6.0	0.2	Extremely narrow	
11	0.5	1.0	Wide	
12	0.1	0.2	Wide	
1	0.2	0.2	Narrow	Steel
2	0.4	0.2	Narrow	
3	0.6	0.2	Narrow	
4	4.0	0.2	Extremely narrow	
5	5.0	0.2	Extremely narrow	
6	6.0	0.2	Extremely narrow	

**Table 7:** Experimental results on the aluminum specimens.

No.	Type of Defect	d/mm	w/mm	RR/μs	RS/μs	Δt/μs	Estimated Depth with Corrected Item	Relative Errors	Estimated Depth without Corrected Item	Relative Errors
1	Aluminum narrow Figure 11a,b	0.2	0.2	8.80	9.04	0.24	0.2009	0.44%	0.4638	131.91%
2		0.3	0.2	8.80	9.08	0.28	0.2782	7.27%	0.5411	80.37%
3		0.4	0.2	8.74	9.09	0.35	0.4135	3.37%	0.6764	69.10%
4		0.5	0.2	8.74	9.14	0.40	0.5101	2.02%	0.7730	54.60%
5		0.6	0.2	8.66	9.10	0.44	0.5874	2.10%	0.8503	41.72%
6	extremely narrow Figure 11e,f	4.0	0.2	8.92	11.09	2.17	3.9307	1.73%	4.1937	4.84%
7		4.5	0.2	8.92	11.24	2.32	4.2206	6.21%	4.4835	0.37%
8		5.0	0.2	8.92	11.44	2.52	4.6071	7.86%	4.8701	2.60%
9		5.5	0.2	8.92	11.64	2.72	4.9936	9.21%	5.2566	4.43%
10		6.0	0.2	8.92	11.96	3.04	5.6121	6.47%	5.8750	2.08%
1	Steel narrow Figure 11c,d	0.2	0.2	7.06	7.30	0.24	0.2009	0.44%	0.4847	142.34%
2		0.4	0.2	7.18	7.53	0.35	0.4135	3.37%	0.7068	76.71%
3		0.6	0.2	7.18	7.65	0.47	0.6454	7.56%	0.9492	58.20%
4	extremely narrow Figure 11g,h	4.0	0.2	7.18	9.18	2.00	3.6022	9.95%	4.0391	0.98%
5		5.0	0.2	7.14	9.60	2.46	4.4912	10.18%	4.9681	0.64%
6		6.0	0.2	6.96	9.80	2.84	5.2255	12.91%	5.7355	4.41%

**Table 8:** Experimental results on the aluminum specimens of wide defects.

No.	d/mm	w/mm	RR/μs	RS/μs	Δt/μs	Estimated Depth with Corrected Item	Relative Errors	Estimated Depth without Corrected Item	Relative Errors
11	0.5	1.0	8.50	9.92	1.42	1.4296	185.91%	2.7442	448.85%
12	0.1	0.2	8.34	8.50	0.16	0.0463	53.72%	0.3092	209.21%

## Data Availability

The processed data required to reproduce the above findings cannot be shared at this time as the data also form part of an ongoing study.
